# A Novel 3D Pedestrian Navigation Method for a Multiple Sensors-Based Foot-Mounted Inertial System

**DOI:** 10.3390/s17112695

**Published:** 2017-11-22

**Authors:** Wei Yang, Chundi Xiu, Jianmin Zhang, Dongkai Yang

**Affiliations:** School of Electronic and Information Engineering, Beihang University, Beijing 100083, China; yangwei89@buaa.edu.cn (W.Y.); zhangjm@buaa.edu.cn (J.Z.); edkyang@buaa.edu.cn (D.Y.)

**Keywords:** indoor positioning, MEMS-IMU, barometer, permanent magnet, zero-velocity update, height estimation, magnetic field strength, extended Kalman filter

## Abstract

In this paper, we present a novel method for 3D pedestrian navigation of foot-mounted inertial systems by integrating a MEMS-IMU, barometer, and permanent magnet. Zero-velocity update (ZUPT) is a well-known algorithm to eliminate the accumulated error of foot-mounted inertial systems. However, the ZUPT stance phase detector using acceleration and angular rate is threshold-based, which may cause incorrect stance phase estimation in the running gait pattern. A permanent magnet-based ZUPT detector is introduced to solve this problem. Peaks extracted from the magnetic field strength waveform are mid-stances of stance phases. A model of peak-peak information and stance phase duration is developed to have a quantitative calculation method of stance phase duration in different movement patterns. Height estimation using barometer is susceptible to the environment. A height difference information aided barometer (HDIB) algorithm integrating MEMS-IMU and barometer is raised to have a better height estimation. The first stage of HDIB is to distinguish level ground/upstairs/downstairs and the second stage is to calculate height using reference atmospheric pressure obtained from the first stage. At last, a ZUPT-based adaptive average window length algorithm (ZUPT-AAWL) is proposed to calculate the true total travelled distance to have a more accurate percentage error (TTDE). This proposed method is verified via multiple experiments. Numerical results show that TTDE ranges from 0.32% to 1.04% in both walking and running gait patterns, and the height estimation error is from 0 m to 2.35 m.

## 1. Introduction

With the rapid development of location-based services (LBSs), multiple pedestrian navigation systems are being widely studied, which can roughly be divided into indoor and outdoor scenarios. Global navigation satellites systems (GNSSs), including GPS, GLONASS, Beidou and GALILEO, are a good method for outdoor navigation [1]. They provide global services and users can get accurate position, velocity and time (PVT) in the open air. However, GNSS signal availability is limited in indoors and urban canyons, so some alternative techniques need to be studied [2].

Several infrastructure-based alternative methods such as WIFI [3,4,5], Bluetooth [6,7], RFID [8], Ultra-Wide Band (UWB) [9,10], LED [11] and ultrasound [12] have been studied over the past years. These methods can provide sufficient positioning accuracy for LBS in normal situations. However, in some special scenarios such as fire emergency relief, infrastructure-based indoor positioning systems are not available. The inertial navigation system (INS) is an infrastructure-free one that uses inertial sensors such as accelerometers and gyroscopes which do not require external reference and installation beforehand [13]. Furthermore, the development of micro-electromechanical system inertial measurement unit (MEMS-IMU) has facilitated the production of inexpensive, lightweight and small-size inertial sensors with low power consumption [14].

MEMS-IMU can provide high accuracy positioning for a short time. However, the drift accumulates very fast, which over time leads to large positioning errors. In order to solve this problem, some researchers have utilized the zero-velocity update (ZUPT) algorithm, which is based on the status of the left and right foot. In other words, the left and right foot alternate between a stationary stance phase and a moving swing phase. The system detects the stance phase and applies ZUPT as pseudo measurements into the extended Kalman filter (EKF) navigation error corrector. This allows the EKF to correct the velocity error during each gait cycle, breaking the cubic-in-time error growth and replacing it with an error accumulation that is linear in the number of steps [15]. Many researchers have improved the ZUPT-aided EKF method. In [16], zero angular rate update (ZARU), heuristic heading reduction (HDR) and electronic compass are used for mitigating heading drift. In [17,18], a module consisting of both hardware and embedded software is provided. In [19], details of how to implement a foot-mounted MEMS-IMU positioning system are described.

The elegance of ZUPT lies in the fact that the foot swings to stance phase periodically during most types of human locomotion, such as walking, running, ascending and descending stairs. The stance phase is the time period during which the ZUPT can be applied to the system with the foot in stationary status [20]. Different ZUPT detectors can be classified into two categories depending on the equipment used. The first kind is using MEMS-IMU alone and the other is integrated with additional devices. MEMS-IMU-based ZUPT detectors utilize acceleration and angular rate which represent the state of motion of the pedestrian directly. Most ZUPT detectors utilize acceleration and angular rate obtained from MEMS-IMU. In [21,22], the stance phase estimation problem is formalized as a binary hypothesis testing problem. Four zero-velocity detectors including the acceleration moving variance detector, the acceleration magnitude detector, the angular rate energy detector, and the stance hypothesis optimal detector are evaluated. In [14,16], three conditions including the magnitude of the acceleration, the local acceleration variance and the magnitude of the gyroscope are used for foot stationary detection. The three conditions must be satisfied simultaneously. The structure of this kind of ZUPT detectors is simple and they have a good stance phase detection accuracy in walking, but when the pedestrian runs, the performance is limited because that this kind of method is threshold-based and the running threshold is not the same compared with walking. The second kind of ZUPT detector uses additional devices such as a shoe-embedded radar [23], a shoe-embedded RF sensor [24], or a shoe-embedded high resolution pressure sensor [25]. Generally speaking, this can bring better performance, but the complexity of system also increases. A permanent magnet-based stance phase detection method was developed in [26]. The authors attached a permanent magnet on one foot and an MEMS-IMU on the other foot. The walking pattern is measured based on the magnetic field strength of the permanent magnet, which changes very rapidly when the foot is in the mid-stance phase. The advantage of a permanent magnetic-based ZUPT detector is that it is not threshold-based and can be applied in both walking and running gait patterns. Compared with other ZUPT detectors using additional devices, mag-ZUPT has a more compact structure in that the measurement device is still a MEMS-IMU and the permanent magnet does not need a power supply. However, stance phase duration is not considered in [26]. Mid-stance should be expanded to stance phase with stance phase duration knowledge. In [27,28,29], the percentage of time involved in the stance is approximately 24.8%. However, we have performed some experiments and found that the stance phase duration is in fact associated with different movement patterns and the 24.8% value is only suitable for walking gait patterns. Stance phase duration is closely related with movement patterns. In order to develop a model of stance phase duration and movement pattern, peak-peak period information obtained from mag-ZUPT is used. Every mid-stance is called one peak and peak-peak period information depicts the frequency of stance phase which is relative with the stance phase duration. Compared with [26], we have a further research of the model of mag-ZUPT. Relationship of peak-peak information and stance phase duration is developed to have a quantitative calculation method of different movement patterns.

The height estimation suffers more from the MEMS-IMU drifts. MEMS-barometers are cheap and useful sensors to be implemented into the MEMS-IMU positioning system [30]. However, the barometer output is heavily influenced by the environment [31,32]. In order to solve this problem, a height difference information-aided barometer (HDIB) algorithm is developed. HDIB is based on the principle that the short time accuracy of INS output is very high and can be used to distinguish the patterns of a person’s vertical movement including walking on the level ground, ascending and descending stairs. This prior information can be used to filter the height estimation results from a barometer.

Percentage error of total travelled distance (TTDE) is used to evaluate the positioning accuracy of INS [33]. It is calculated from positioning error and total travelled distance in closed paths. However, because that the sampling frequency of MEMS-IMU is very high (usually more than 50 Hz), the trajectory calculated from the pedestrian navigation system is the trace of the foot which is mounted with MEMS-IMU. The length of this trajectory is longer than the actual trajectory which leads to the result that TTDE is smaller than the reality. In this paper, we develop a ZUPT based adaptive average window length method (ZUPT-AAWL). It is a more accurate and universal total travelled distance calculation method.

In conclusion we explore three questions in this paper:

- How to use the permanent magnet in running movement to get a more accurate ZUPT estimation?
- How to get a more accurate height estimation fusing MEMS-IMU and barometer?
- How to get a more accurate TTDE?

**Figure 1:**
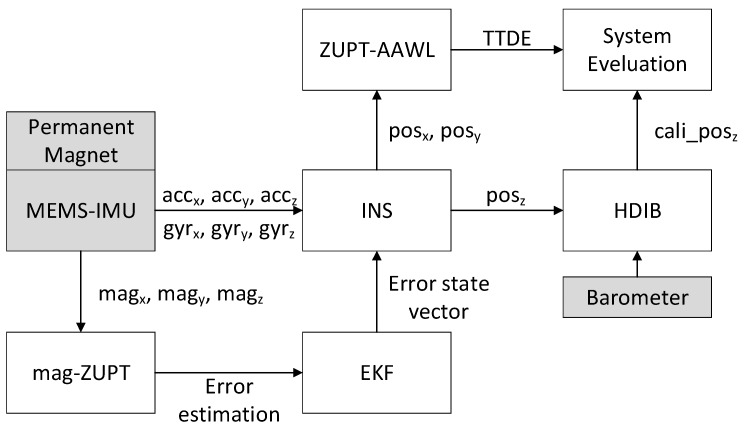
Structure of this novel 3D positioning system.

In order to solve these problems, we developed a novel 3D pedestrian navigation method integrating MEMS-IMU, barometer and permanent magnet which is shown in Figure 1.

This system contains three sensors including one MEMS-IMU, one barometer and one permanent magnet. In fact, magnetometer is not part of IMU. Considering that usually accelerometer, gyroscope and magnetometer are integrated in one device, magnetometer is regarded as part of MEMS-IMU in this paper for convenience. MEMS-IMU collects the 3-axis accelerometer, gyroscope and magnetometer readings which are $\left(a_{x}^{b},a_{y}^{b},a_{z}^{b}\right)$, $\left(\omega_{x}^{b},\omega_{y}^{b},\omega_{z}^{b}\right)$ and $\left(mag_{x}^{b},mag_{y}^{b},mag_{z}^{b}\right)$, respectively. The magnetometer readings are the composite magnetic field strength of the earth magnetic field and the permanent magnet which is used to detect the mid-stance phase and then expanded to the stance phase. Unlike acceleration and angular rate, air pressure value obtained from barometer is only one dimension.

The basic structure of a foot-mounted MEMS-IMU positioning system is INS-EKF-ZUPT (IEZ). Stance phase information obtained from mag-ZUPT is used to get the error estimation and transmitted to EKF to calculate error state vector. INS calculates the positioning results (*pos_x_*,*pos_y_*,*pos_z_*). (*pos_x_*,*pos_y_*) is the planar positioning result and *pos_z_* is the vertical positioning result. In order to get a better height estimation, a barometer is used in this system. The difference information of *pos_z_* can distinguish the patterns of the person’s vertical movement including walking on the level ground, ascending and descending stairs. This information can be used to filter the height estimation from the barometer to get a better height positioning result.

TTDE is an important indicator to evaluate positioning accuracy. In order to get an accurate TTDE, the total travelled distance must be correctly calculated. ZUPT information is used to estimate the average window length and this novel method is called ZUPT-based adaptive average window length method (ZUPT-AAWL).

The reminder of this paper is organized as follows: in Section 2, our MEMS-IMU-based foot-mounted inertial navigation system is described. In Section 3, a novel permanent magnet-based zero-velocity detector (mag-ZUPT) is described. In Section 4, a height difference information aided barometer (HDIB) algorithm is provided. In Section 5, a mag-ZUPT-based adaptive average window length method (ZUPT-AAWL) is provided. In Section 6, experiments on different trajectories are undertaken and the performance is verified. Finally, Section 7 concludes this work and offers some future research suggestions.

## 2. MEMS-IMU Based Foot-Mounted Inertial Navigation System

### 2.1. Inertial Navigation System (INS)

**Figure 2:**
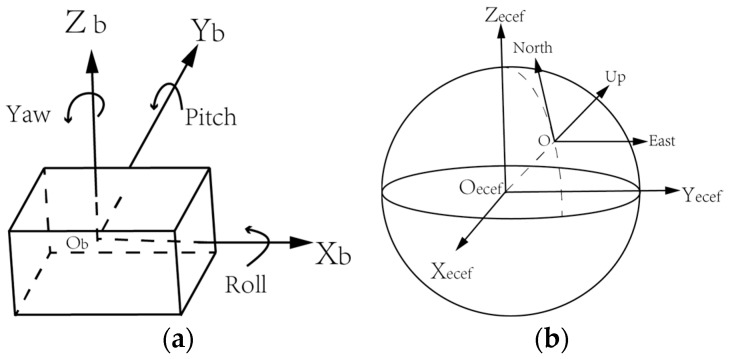
Sketch map of sensor body coordinate frame (b-frame) and navigation coordinate frame (n-frame). (**a**) Shows the b-frame and three Euler angels which are yaw, pitch and roll; (**b**) shows the relation between earth-centered, earth fixed (ECEF) coordinate system and east-north-up (ENU) coordinate system. ENU is better to be applied in our system than ECEF because that it has the same origin with b-frame. Therefore, the rotation matrix will be simpler.

A foot-mounted INS uses 3-axis accelerations and angular rates to calculate the position of pedestrians. The principle of INS is that the integration of acceleration is velocity and the integration of velocity is position. The outputs of MEMS-IMU are in the sensor body coordinate frame (b-frame) and should be transferred to the navigation coordinate frame (n-frame). The definition of b-frame and n-frame are shown in Figure 2.

The b-frame is determined by the MEMS-IMU hardware and usually defined as a right-handed Cartesian coordinate system, which is shown in Figure 2a. The forward, left and upward direction are *x*, *y* and *z* axis, respectively. Considering the convenient of usage, the n-frame applied in our system is the local East, North, Up (ENU) Cartesian coordinate system whose origin is the same with b-frame which is more intuitive and practical than ECEF or Geodetic coordinates. By convention, the east axis is labelled *x*, the north *y* and the up *z*. ECEF and ENU are depicted in Figure 2b. The MEMS-IMU readings including acceleration, angular rate and magnetic field strength are in b-frame and should be transferred to ENU in order to derive the velocity and the position. The two different coordinate systems are transferred through the rotation matrix $C_{b}^{n}$. It is defined from yaw, pitch and roll:

(1) $$C_{b}^{n}=\left[\begin{array}{ccc} \cos\theta \cos\psi & -\cos\gamma \sin\psi +\sin\gamma \sin\theta \cos\psi & \sin\gamma \sin\psi +\cos\gamma \sin\theta \cos\psi \\ \cos\theta \sin\psi & \cos\gamma \cos\psi +\sin\gamma \sin\theta \sin\psi & -\sin\gamma \cos\psi +\cos\gamma \sin\theta \sin\psi \\ -\sin\theta & \sin\gamma \cos\theta & \cos\gamma \cos\theta \end{array}\right]$$

where ψ is yaw, θ is pitch and γ is roll. $C_{b}^{n}$ should be initialized using accelerometer measurements before one pedestrian starts to walk. In the rotation matrix initialization step, MEMS-IMU remains stable for several seconds and the accelerations measured in the *x*, *y* and *z* axes of b-frame are $\left[a_{x}^{b},a_{y}^{b},a_{z}^{b}\right]$, and the gravity of the n-frame is [0,0,g]. Therefore, the initialization equation is:

(2) $$\left[\begin{array}{l} a_{x}^{b}\\ a_{y}^{b}\\ a_{z}^{b} \end{array}\right]={\left(C_{b}^{n}\right)}^{T}\left[\begin{array}{l} 0\\ 0\\ g \end{array}\right]$$

The calculated result of Equation (2) is:

(3) $$\left\{ \begin{array}{l} \gamma_{0}=\arctan(\frac{a_{y}^{b}}{a_{z}^{b}})\\ \theta_{0}=\arctan(\frac{a_{x}^{b}}{\sqrt{{\left(a_{y}^{b}\right)}^{2}+{\left(a_{z}^{b}\right)}^{2}}}) \end{array}\right.$$

where $\gamma_{0}$ is initial roll and $\theta_{0}$ is initial pitch. We can’t get initial yaw ($\psi_{0}$) using accelerations alone. In some researches, $\psi_{0}$ is manually set to a constant like 0. Magnetometer measurements are used to obtain $\psi_{0}$ in this paper and the magnetometer have been calibrated to the local geomagnetic field.

(4) $$\psi_{0}=\arctan(\frac{mag_{z}^{b}\sin(\gamma_{0})-mag_{y}^{b}\cos(\gamma_{0})}{mag_{x}^{b}\cos(\theta_{0})+mag_{y}^{b}\sin(\theta_{0})\sin(\gamma_{0})+mag_{z}^{b}\sin(\theta_{0})\cos(\gamma_{0})})$$

where $mag_{x}^{b},\;mag_{y}^{b}$, and $mag_{z}^{b}$ are the *x*-axis, *y*-axis and *z*-axis magnetometer measurements of the b-frame, respectively.

The 3-axis gyroscope makes it possible to update $C_{b}^{n}$. Integrating the angular rates gives the relative orientation of the IMU [16]:

(5) $$C_{b}^{n}(k)=C_{b}^{n}(k-1)\cdot \frac{2I_{3\times 3}+\Omega (k)}{2I_{3\times 3}-\Omega (k)}$$

where Ω(*k*) is:

(6) $$\Omega (k)=\left[\begin{array}{ccc} 0 & -\omega_{z}(k) & \omega_{y}(k)\\ \omega_{z}(k) & 0 & -\omega_{x}(k)\\ -\omega_{y}(k) & \omega_{x}(k) & 0 \end{array}\right]$$

where $\omega_{x},\;\omega_{y}$ and $\omega_{z}$ are the *x*-axis, *y*-axis and *z*-axis of the b-frame gyroscope measurements, respectively. Finally, $C_{b}^{n}$ should be corrected according to the error state vector from the EKF process. It will be explained in the INS error model and the extended Kalman filter part of this paper.

The 3-axis gyroscope makes it possible to calculate $C_{b}^{n}$. Integrating the angular rates gives the relative orientation of the IMU, which can be used to transform the accelerometer measurement [18].

Euler angles, direction cosines and quaternions are the three most used rotation matrix parametrization methods. The reasons why quaternions are better to be used in navigation applications is:

- Quaternions only need to solve four equations, while direction cosines need to solve six differential equations. Therefore, the computational complexity of quaternions is smaller and this advantage is in favor of a real-time navigation system.
- Euler angles would bring singular values when the angles are ±90°. Quaternions do not have such a problem.

The quaternions were first developed by Irish mathematician William Rowan Hamilton in 1843. Quaternions are very useful to calculate three-dimensional rotations and unit quaternions can represent any rotation operation in three-dimensional space. Quaternions are a number system that extends the complex numbers and usually represented in the following form:

(7) $$q=q_{0}+q_{1}i+q_{2}j+q_{3}k$$

where $q_{0},\;q_{1},\;q_{2}$ and $q_{3}$ are real numbers. *i, j* and *k* are fundamental quaternion units. The differential equation of quaternion is:

(8) $$q(k)=\{\cos\frac{\Delta \theta_{0}}{2}I+\frac{\left[\Delta \theta \right]}{\Delta \theta_{0}}\sin\frac{\Delta \theta_{0}}{2}\}q(k-1)$$

where $\Delta \theta_{x},\;\Delta \theta_{y},\;\Delta \theta_{z}$ $\mathrm{and}\;\Delta \theta_{0}$ are defined as:

(9) $$\{\Delta \theta_{x},\Delta \theta_{y},\Delta \theta_{z}\}=\{\omega_{x}^{\prime }(k),\omega_{y}^{\prime }(k),\omega_{z}^{\prime }(k)\}\cdot \Delta t$$

(10) $$\Delta \theta_{0}=\sqrt{\Delta \theta_{x}^{2}+\Delta \theta_{y}^{2}+\Delta \theta_{z}^{2}}$$

and Δθ is:

(11) $$\Delta \theta =\left[\begin{array}{cccc} 0 & -\Delta \theta_{x} & -\Delta \theta_{y} & -\Delta \theta_{z}\\ \Delta \theta_{x} & 0 & \Delta \theta_{z} & -\Delta \theta_{y}\\ \Delta \theta_{y} & -\Delta \theta_{z} & 0 & \Delta \theta_{x}\\ \Delta \theta_{z} & \Delta \theta_{y} & -\Delta \theta_{x} & 0 \end{array}\right]$$

The rotation matrix can be transferred to the quaternion form:

(12) $$C_{b}^{n}=\left[\begin{array}{ccc} q_{0}^{2}+q_{1}^{2}-q_{2}^{2}-q_{3}^{2} & 2(q_{1}q_{2}-q_{0}q_{3}) & 2(q_{1}q_{3}+q_{0}q_{2})\\ 2(q_{1}q_{2}+q_{0}q_{3}) & q_{0}^{2}-q_{1}^{2}+q_{2}^{2}-q_{3}^{2} & 2(q_{2}q_{3}-q_{0}q_{1})\\ 2(q_{1}q_{3}-q_{0}q_{2}) & 2(q_{2}q_{3}+q_{0}q_{1}) & q_{0}^{2}-q_{1}^{2}-q_{2}^{2}+q_{3}^{2} \end{array}\right]$$

and we can calculate the four real numbers of quaternion from $C_{b}^{n}$ using:

(13) $$\{\begin{array}{c} q_{0}=\frac{\sqrt{1+C_{b}^{n}(1,1)+C_{b}^{n}(2,2)+C_{b}^{n}(3,3)}}{2}\\ q_{1}=\frac{C_{b}^{n}(2,3)-C_{b}^{n}(3,1)}{4q_{0}}\\ q_{2}=\frac{C_{b}^{n}(3,1)-C_{b}^{n}(1,3)}{4q_{0}}\\ q_{3}=\frac{C_{b}^{n}(1,2)-C_{b}^{n}(2,1)}{4q_{0}} \end{array}$$

**Figure 3:**
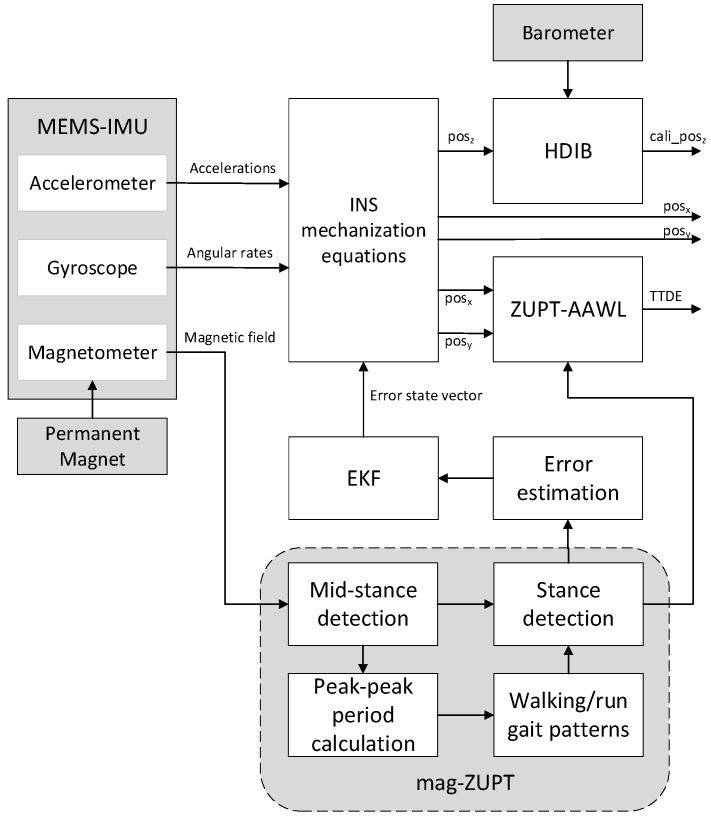
Flowchart of the pedestrian navigation system with EKF and ZUPT.

Figure 3 shows the flowchart of the pedestrian navigation system with EKF and ZUPT. HDIB, ZUPT-AAWL and mag-ZUPT described in this paper is also shown in this figure. The pedestrian navigation system mainly contains three parts which are INS mechanization equations, EKF for error estimation and ZUPT.

INS mechanization equations are a set of equations which form the basis of inertial navigation. Firstly, gravity should be subtracted from accelerometer measurements in n-frame. Then position is calculated with the gravity-free acceleration value. At last, orientation of the MEMS-IMU is updated with the gyroscope measurements. These equations take slight different forms in different navigation frames [34]. The basic equation utilizing accelerometers and gyroscopes to calculate position is [17]:

(14) $$\left[\begin{array}{c} \begin{array}{c} v_{k}\\ p_{k} \end{array}\\ q_{k} \end{array}\right]=\left[\begin{array}{c} \begin{array}{c} v_{k-1}+(q_{k-1}a_{k}q_{k-1}^{-1}-g)dt_{k}\\ p_{k-1}+v_{k-1}dt_{k} \end{array}\\ \Omega (\omega_{k}dt_{k})q_{k-1} \end{array}\right]$$

where *k* is a time index, g is the gravity, $v_{k}$ is the velocity of the pedestrian, $a_{k}$ is the accelerometer measurements, $p_{k}$ is the positon of the person, $q_{k}$ is the quaternion describing the orientation frame, dt is the time differential and Ω(·) is the quaternion update matrix.

### 2.2. INS Error Model and the Extended Kalman Filter (EKF)

The state vector of this system is a 15-element vector which is δx=[δr,δv,δφ,δa,δω], where δr is the position error, δv is the velocity bias, δφ is the attitude error, δa is the accelerometer bias, δω is the gyroscope error. In addition, δr,δv,δφ,δa,δω are all 3-dimension vectors.

The state transition matrix *F* and *G* are:

(15) $$F=\left[\begin{array}{ccccc} I & I\cdot \Delta t & O & O & O\\ O & I & St\cdot \Delta t & C_{b}^{n}\cdot \Delta t & O\\ O & O & I & O & -C_{b}^{n}\cdot \Delta t\\ O & O & O & I & O\\ O & O & O & O & I \end{array}\right]$$

(16) $$G=\left[\begin{array}{cccc} O & O & O & O\\ C_{b}^{n}\cdot \Delta t & O & O & O\\ O & -C_{b}^{n}\cdot \Delta t & O & O\\ O & O & I & O\\ O & O & O & I \end{array}\right]$$

where *Δt* is the sample interval, *O* and *I* are:

(17) $$O=\left[\begin{array}{ccc} 0 & 0 & 0\\ 0 & 0 & 0\\ 0 & 0 & 0 \end{array}\right]$$

(18) $$I=\left[\begin{array}{ccc} 1 & 0 & 0\\ 0 & 1 & 0\\ 0 & 0 & 1 \end{array}\right]$$

*St* is the skew-symmetric matrix of acceleration:

(19) $$St=\left[\begin{array}{ccc} 0 & -a_{z}(k) & a_{y}(k)\\ a_{z}(k) & 0 & -a_{x}(k)\\ -a_{y}(k) & a_{x}(k) & 0 \end{array}\right]$$

The measurement model is:

(20) z(k)=−v(k)

where v(k) is the velocity when ZUPT detects that the person is in stance phase:

(21) $$H=\left[\begin{array}{ccccc} O & I & O & O & O \end{array}\right]$$

The algorithm has the following steps:

(1) The acceleration and angular rate are compensated according to the acceleration and angular rate bias output of EKF:
  (22) $$a^{\prime }(k)=a^{\prime }(k)+\delta a(k-1)$$
  (23) $$\omega^{\prime }(k)=\omega^{\prime }(k)+\delta \omega (k-1)$$
  where *a*’(*k*) and *ω*’(*k*) are the compensated acceleration and angular rate at the time index *k*. *a*(*k*) and *ω*(*k*) are the original acceleration and angular rate at time index *k*. *δa*’(*k−*1) and *δω*(*k−*1) are the acceleration and angular rate error from EKF at time index *k* − 1.
(2) Update the quaternion:
  (24) $$q(k)=\{\cos\frac{\Delta \theta_{0}}{2}I+\frac{\left[\Delta \theta \right]}{\Delta \theta_{0}}\sin\frac{\Delta \theta_{0}}{2}\}q(k-1)$$
  where $\Delta \theta_{0}={\left|\omega^{\prime }\left(k\right)\right|}^{2},\;\Delta \theta$ is:
  (25) $$\Delta \theta =\left[\begin{array}{cccc} 0 & -{\omega^{\prime }}_{x}(k) & -{\omega^{\prime }}_{y}(k) & -{\omega^{\prime }}_{z}(k)\\ {\omega^{\prime }}_{x}(k) & 0 & {\omega^{\prime }}_{z}(k) & -{\omega^{\prime }}_{y}(k)\\ {\omega^{\prime }}_{y}(k) & -{\omega^{\prime }}_{z}(k) & 0 & {\omega^{\prime }}_{x}(k)\\ {\omega^{\prime }}_{z}(k) & {\omega^{\prime }}_{y}(k) & -{\omega^{\prime }}_{x}(k) & 0 \end{array}\right]$$
  where ${\omega^{\prime }}_{x}\left(k\right),\;{\omega^{\prime }}_{y}\left(k\right)$ and ${\omega^{\prime }}_{z}\left(k\right)$ are the three components of $\omega^{\prime }\left(k\right)$ in the *x*, *y* and *z* axis, respectively.
(3) Velocity and position update:
  (26) $$v(k)=v(k-1)+a_{n}(k)\cdot \Delta t$$
  (27) $$r(k)=r(k-1)+v(k)\cdot \Delta t+\frac{1}{2}a_{n}(k)\cdot \Delta t^{2}$$
  where *k* is the time index, *v*(*k*) is the velocity and *r*(*k*) is the position, *a_n_*(*k*) is the original acceleration transferred to the navigation-frame.
(4) Correct rotation matrix $C_{b}^{n}$. δφ is used to compensate $C_{b}^{n}$:
  (28) $$C_{b}^{n^{\prime }}(k)=\frac{2I_{3\times 3}+\delta \Theta (k)}{2I_{3\times 3}-\delta \Theta (k)}\cdot C_{b}^{n}(k)$$
  where δΘ is:
  (29) $$\delta \Theta =\left[\begin{array}{ccc} 0 & -\delta \varphi (3) & \delta \varphi (2)\\ \delta \varphi (3) & 0 & -\delta \varphi (1)\\ -\delta \varphi (2) & \delta \varphi (1) & 0 \end{array}\right]$$
  where δφ(3), δφ(2) and δφ(1) are the three components of δφ from EKF which are the yaw, pitch and roll errors, respectively. $C_{b}^{n}\left(k\right)$ and $C_{b}^{n\prime }\left(k\right)$ are the attitude matrix before and after compensation. *I* is the 2-dimension unitary matrix.
(5) Compensate velocity and position using the error measurements from EKF:
  (30) $$r^{\prime }(k)=r(k)+\delta r(k-1)$$
  (31) $$v^{\prime }(k)=v(k)+\delta v(k-1)$$
  where *r’*(*k*) is the compensated position and *v’*(*k*) is the compensated velocity. δr(k−1) and δv(k−1) are position and velocity error at time index *k* − 1.

## 3. A Novel Stance Phase Duration Model of Permanent Magnet Based ZUPT Detector (Mag-ZUPT)

### 3.1. Disadvantages of Threshold Based Stance Phase Detector in Running

There is a basic phenomenon that when the pedestrian’s foot is totally on the ground, the velocity and angular velocity are almost zero. Considering that the positioning errors will accumulate fast due to the sensor drift, the zero-velocity information is efficient in the error correction. The detection problem is a binary hypothesis testing problem. All the ZUPT methods are based on the MEMS-IMU information including the accelerometer and gyroscope readings [22]. It can be depicted by:

(32) T=f(a,ω) ≤ γ

where *a* is the acceleration, ω is the angular rate, *γ* is the stance phase threshold. *T* is the test statistics of the detector. One typical detector is the stance hypothesis optimal detector (SHOE):

(33) $$T(k)=\frac{1}{W}\sum_{n=k}^{k+W-1}\frac{1}{\sigma_{a}^{2}}{\left\|a(n)-g\cdot \frac{\bar{a(k)}}{\left\|\bar{a(k)}\right\|}\right\|}^{2}+\frac{1}{\sigma_{w}^{2}}{\left\|w(n)\right\|}^{2}\leq \gamma$$

where *k* is a time index, *W* is the window length, $\sigma_{a}^{2}$ and $\sigma_{w}^{2}$ denote the accelerometer and gyroscope noise variance, ‖⋅‖ is the 2-norm calculation, $\bar{a\left(k\right)}$ is the average of *a* during the average window *W* at time index *k*:

(34) $$\bar{a(k)}=\frac{1}{W}\sum_{n=k}^{k+W-1}a(n)$$

**Figure 4:**
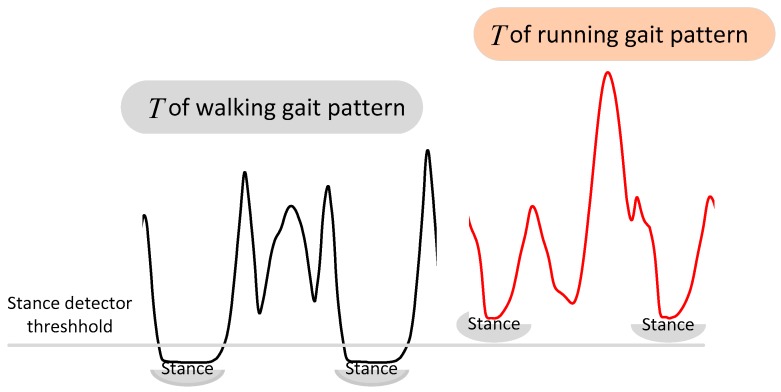
Comparison of *T* in a walking and running gait pattern.

This ZUPT detector is efficient in most walking scenarios. However, this method is threshold- based and it may not be suitable when the pedestrian runs. Figure 4 shows the comparison of *T* in walking and running gait patterns. It is obvious that *T* of a running gait pattern is larger than when walking and the stance detector suitable for walking is not a proper choice for running. Therefore, if the pedestrian moves in a hybrid pattern including walking and running, there may be a wrong stance detection of running which will lead to a large accumulative error.

**Figure 5:**
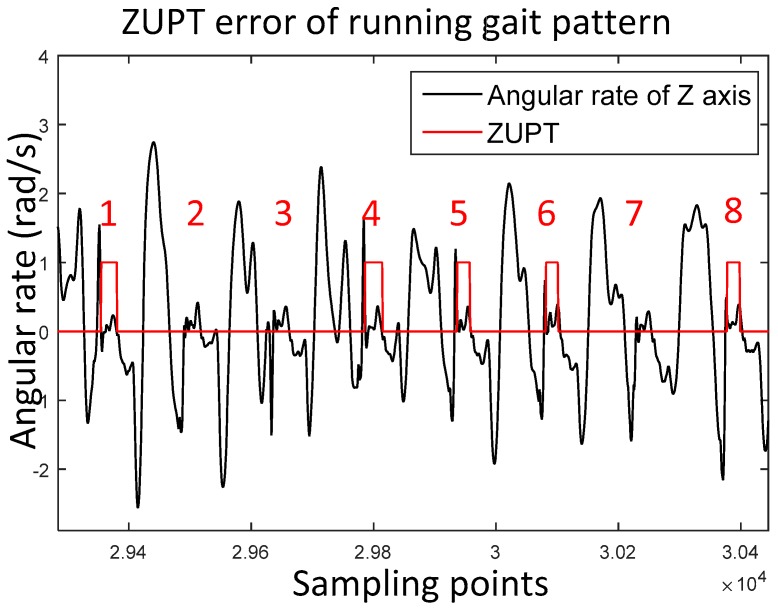
Stance phase detection errors of running gait pattern. The black wave contains eight running gait cycles. The red line represents ZUPT results in which 1 is the stance phase and 0 is the swing phase. The numbers from 1 to 8 are the identifier of the gait cycle.

The stance phase estimation error of running gait pattern is shown in Figure 5. It is clear that the No. 2, 3 and 7 stance phases are not detected.

### 3.2. Mid-Stance Detection Method with a Permanent Magnet Moutned on the Foot

**Figure 6:**
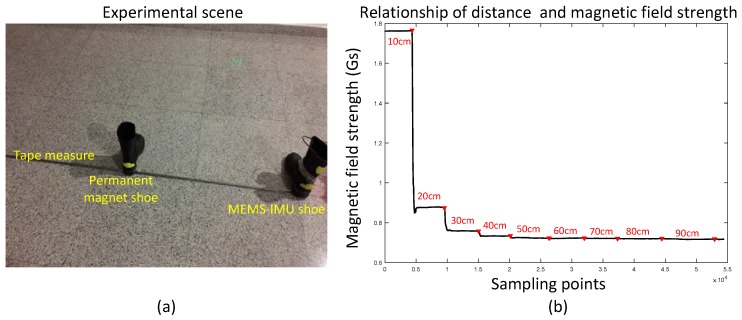
Relationship of distance and magnetic field strength; (**a**) is a picture of the experiment scene. The left shoe is mounted with a MEMS-IMU and the right shoe is mounted with a permanent magnet. The right shoe moves along the ruler and stops every 10 cm; (**b**) is the result of the relationship between distance and magnetic field strength.

It is important to research a new stance phase detector which is more robust and can be applied in both walking and running gait patterns. In [26] a permanent magnet is used as an additional device to have a better stance phase detection. One permanent magnetic detector is attached to the MEMS-IMU free foot to create a local magnetic field and from which the mid-stance can be detected. The basic principle is about the relationship of magnetic field strength and distance. It is obvious that the magnetic field strength is stronger when the distance between magnet and sensor decrease. An experiment was taken to analyze this phenomenon and the result is shown in Figure 6.

**Figure 7:**
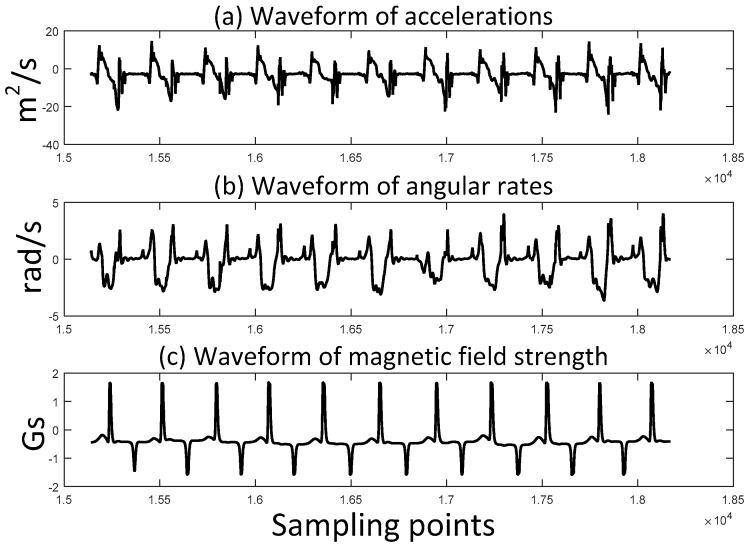
Waveforms of accelerations, angular rates and magnetic field strength during the same time period of walking gait pattern.

**Figure 8:**
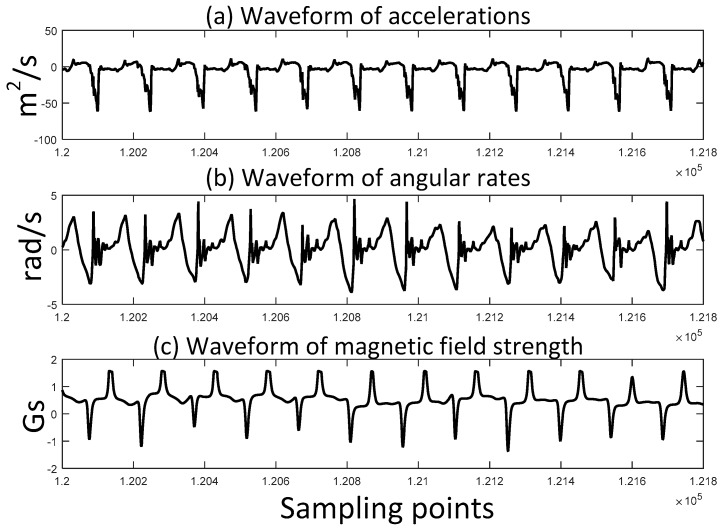
Waveforms of accelerations, angular rates and magnetic field strength during the same time period of running gait pattern.

One of the advantages of applying a permanent magnet is that the magnetic field strength is smoother than the acceleration and angular rate. Figure 7 and Figure 8 show the comparison of the waveforms of accelerations, angular rates and magnetic field strength in walking and running gait patterns, respectively.

**Figure 9:**
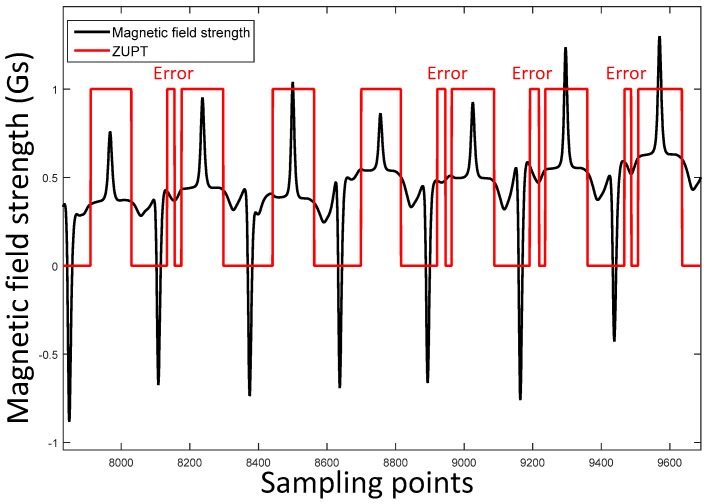
ZUPT errors and the magnetic field strength waveform.

It is clear that the waveform of magnetic field strength contains less noise than acceleration and angular rate. Furthermore, in the running gait pattern, the waveform of acceleration becomes worse while magnetic field strength remains steady. The fluctuation of acceleration and angular rate leads to wrong estimation of stance phase which is shown in Figure 9. The stance phase is misjudged to swing phase, while the mid-stance can be correctly detected. The magnetic field strength of the permanent magnet is useful to develop a better ZUPT detector.

**Figure 10:**
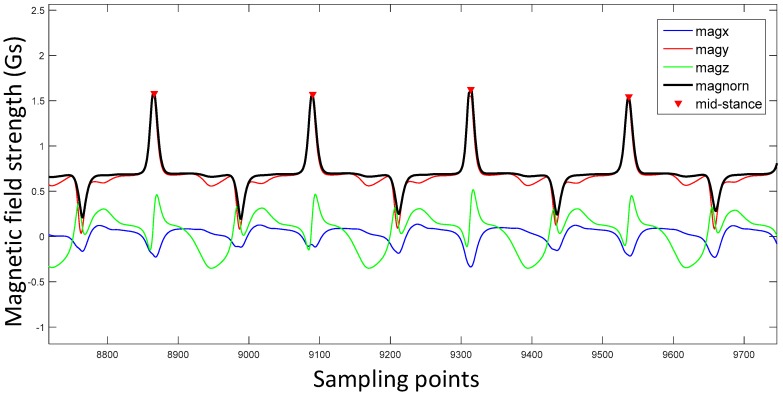
Comparison of different magnetic field strength waveforms. The blue line, red line, green line and black line are *magx*, *magy*, *magz* and *magnorm*, respectively. Mid-stances (peaks) from *magnorm* are marked with red triangles.

Mid-stances can be extracted from peaks of magnetic field strength waveforms which are closely related with the placing relation of MEMS-IMU and the permanent magnet. In this system, the norm of (magx,magy,magz) is used:

(35) $$magnorm=\sqrt{magx^{2}+magy^{2}+magz^{2}}$$

where *magnorm* is the norm of (*magx,magy,magz*). The comparison of different magnetic field waveforms is shown in Figure 10. From this figure we can conclude that the norm of (*magx,magy,magz*) is the best mid-stance indicator and magy has a similar performance while *magx* and *magz* are not proper choices. There are many methods to detect the peaks of *magnorm* such as utilizing the gradient of the waveforms. The peaks are also shown in Figure 10 with red triangles.

### 3.3. Mag-ZUPT Method with a Stance Phase Duration Model

**Figure 11:**
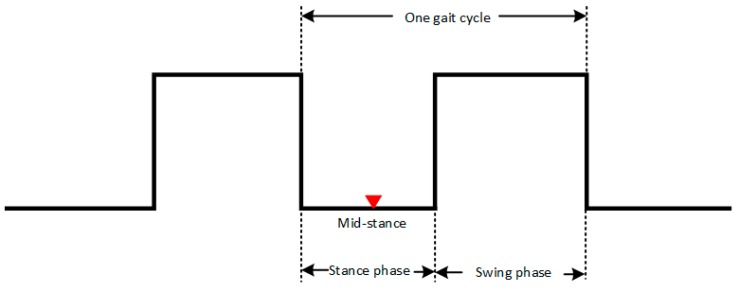
Sketch map of one gait cycle. It contains stance phase and swing phase. Paper [25] only shows how to detect mid-stance. We expend mid-stance to stance phase using stance phase duration information.

Mid-stance can be extracted from the magnetic field strength waveform of the permanent magnet. However, mid-stance is only a point of the stance phase. The sketch map of one gait cycle is shown in Figure 11. One gait cycle can be divided into stance phase and swing phase. Mid-stance is a time point when the foot is totally on the ground.

**Figure 12:**
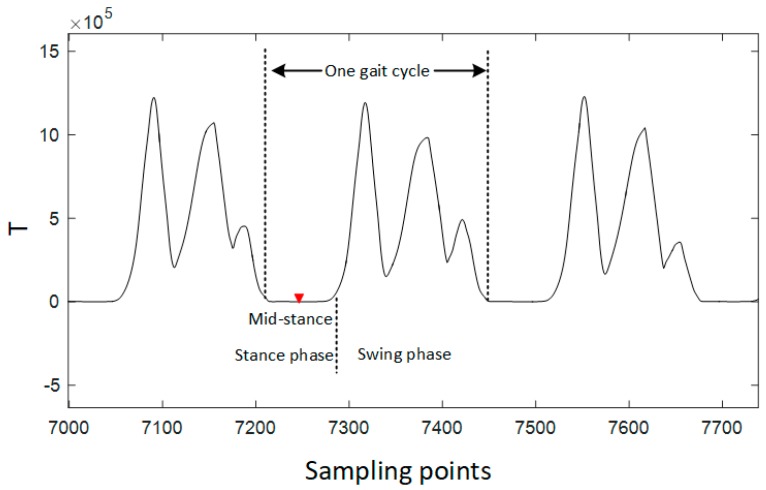
Relationship of mid-stance, stance phase, swing phase and gait cycle. Mid-stance can be expanded to stance phase using stance phase duration information. *T* is the test statics of SHOE detector.

An experiment was taken to have a more clear clarification of the components in one gait cycle which is shown in Figure 12. *T* is the test statistics of the SHOE detector calculated from Equation (33). With the information of the time duration of stance phase in one gait cycle, each mid-stance can be expanded to a stance phase. According to former researches, the duration of the stance phase is about 24.8%. However, the duration of stance phase is related with the different moving patterns. If 24.8% is used in the running gait pattern, the total travelled distance will be smaller than the reality because that some swing phases are misjudged to stance-phase.

**Figure 13:**
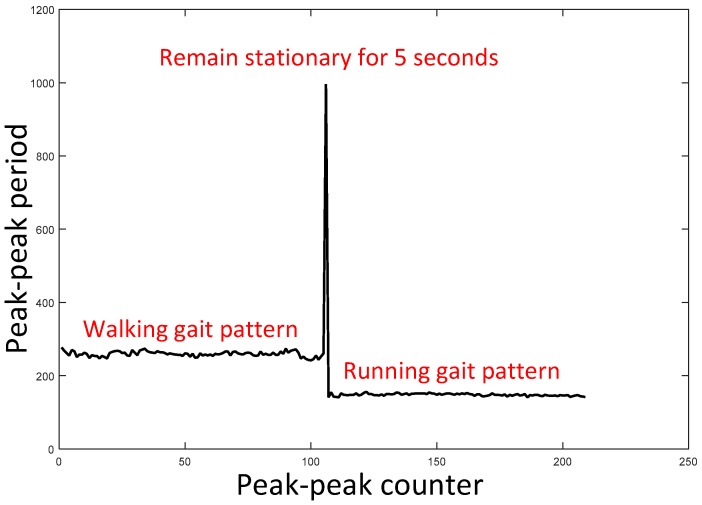
Using peak-peak time period information to distinguish walking/running gait pattern.

The two typical types of a pedestrian indoor are walking and running. Considering that the length between the neighbor mid-stances is one complete gait cycle, the gait cycle length of the same person in one trajectory is directly related with the moving pattern. Figure 13 depicts the peak-peak time period of walking, standing still and running. This experiment is taken in a designed scene: (1) Firstly a person walks for 140 s; (2) Secondly, the person remains stationary for 5 s; (3) At last, the person runs for 80 s. The statistic result is shown in Table 1. The maximum of running peak-peak time period is even smaller than the minimums of walking peak-peak time period. Therefore, peak-peak time period is a reliable method to distinguish walking and running gait patterns.

A simple stance phase duration model is proposed. The relationship between peak-peak period and stance phase duration is:

(36) $$spd(k)=\frac{1}{1000N}\sum_{n=k}^{k+N-1}pp(k)$$

where *k* is a time index, *N* is the window length, *spd* is stance phase duration and *pp* is the peak-peak period. For example, referring to the experiment of Figure 13 and Table 1, the stance phase durations of walking and running gait patterns are 25.9% and 14.8%. These two values are acceptable in walking/running mix trajectories. In fact, the relationship between movement patterns and stance phase duration is complex. We should consider many factors like body height and leg length. In the next paper, we will develop a more sophisticated model using big data trained by artificial neural network. In addition, a random forest based moving pattern estimation method will also be described.

**Figure 14:**
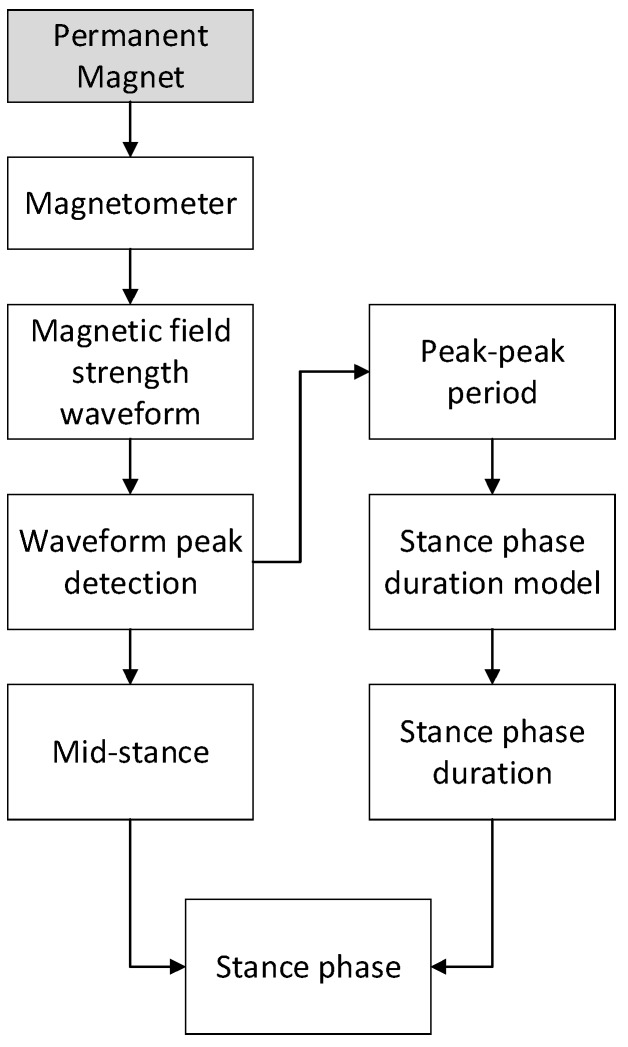
Flowchart of mag-ZUPT. Mid-stance (peak) is extracted from magnetic field strength waveforms. Peak-peak period is used to distinguish walking/running gait patterns and then to obtain stance phase duration. Finally, mid-stance is expanded to stance phase.

The flowchart of how to detect mid-stance and how to expand mid-stance to stance phase is shown in Figure 14. The mag-ZUPT algorithm contains three steps. The first step is to extract mid-stance from magnetic field waveforms. The second step is to distinguish walking/running gait patterns using peak-peak period. Finally, mid-stance is expanded to stance phase with stance phase duration.

**Figure 15:**
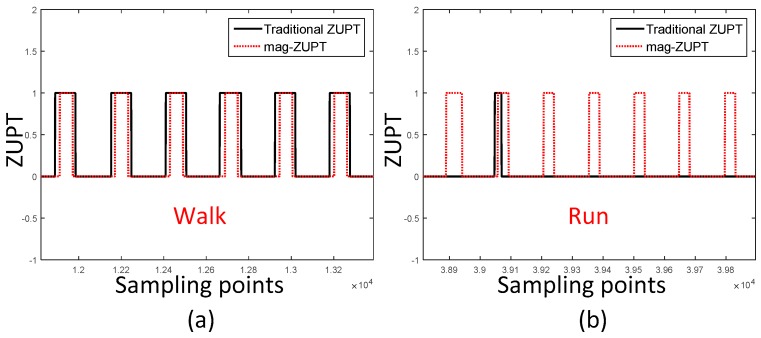
Traditional ZUPT and mag-ZUPT results in walking and running gait patterns; (**a**) is the walking scenario. The two ZUPT detectors have a similar performance; (**b**) is the running scenario. Seven stance phases are correctly detected by mag-ZUPT while only one stance phase is detected by traditional ZUPT.

Like other ZUPT detectors, stance phase is represented as 1 and swing phase is represented as 0 to compose mag-ZUPT. The results of traditional ZUPT and mag-ZUPT is shown in Figure 15. The two ZUPT detectors have a similar performance in walking. While as to the running gait pattern, seven stance phases are correctly detected by mag-ZUPT compared with only one stance phase is detected by traditional ZUPT. Therefore, mag-ZUPT has a better performance especially in running scenarios.

**Figure 16:**
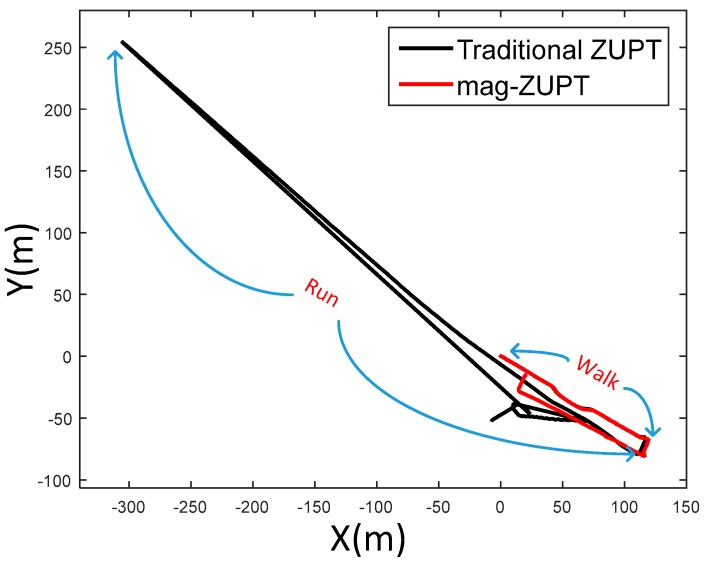
Positioning results of a walking/running mix trajectory. The first half path is walking and the second half path is running. Traditional ZUPT cannot detect stance phase correctly of running gait pattern, therefore the positioning result diverges rapidly.

An experiment of a walking/running mix trajectory was undertaken to verify the performance of the mag-ZUPT method. The trajectory is a closed path that the first half of the trajectory is walking and then the pedestrian ran back to the start point. The positioning results using mag-ZUPT and traditional ZUPT are shown in Figure 16. It is obvious that the walking trajectory (first half) of these two methods are similar. However, the running trajectory using traditional ZUPT diverges rapidly because of the stance phase estimation errors.

## 4. Height Difference Information Aided Barometer (HDIB) Algorithm

**Figure 17:**
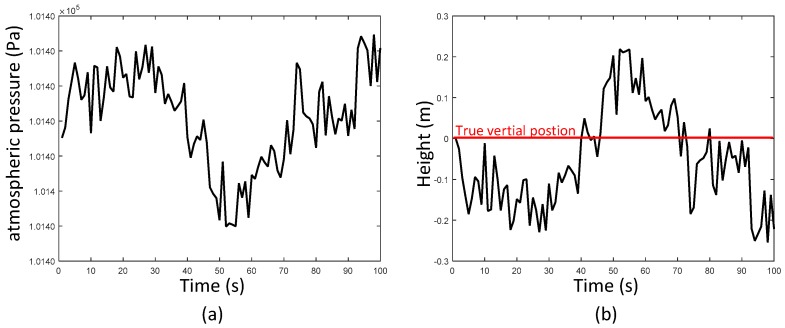
The atmospheric pressure fluctuates when the pedestrian walks on the same height; (**a**) is the atmospheric pressure; (**b**) is the height estimation error caused by atmospheric pressure fluctuation. The red line in (**b**) is the true vertical position (0 m).

Considering that a bias of only a few meters can lead to an error of several floors, the demand of height accuracy is even higher than the level ground scenario. However, the accelerometer and the gyroscope drifts are accumulated to obtain a poor height estimation. As to the barometer, it is easy to be influenced by the environment and this phenomenon is shown in Figure 17. An experiment was taken that the pedestrian walked on the level ground for 100 s and it is obvious that even on the level ground, the atmospheric pressure fluctuates with the pedestrian movement which leads to height estimation errors.

**Figure 18:**
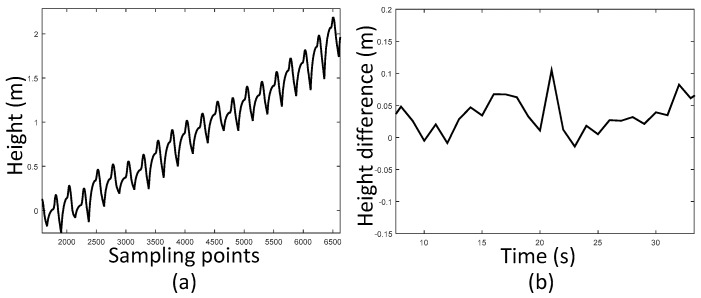
The accumulative error of height estimation and the height difference every one second; (**a**) is the accumulative error. It reaches 2 m after 5000 sampling points (25 s); (**b**) is the height difference every one second. Although the height error accumulated, the height difference stay steady and it is possible to use this information to aid barometer to estimate the height more precisely.

In order to achieve a high accuracy height estimation in our 3D positioning system, a novel height difference information aided barometer (HDIB) algorithm is developed. HDIB utilizes both MEMS-IMU and barometer readings. It is dependent on the basic thinking that normally the height motion of a person is determined by the ground mode which includes level ground, upstairs and downstairs. When walking on a level ground, the height is invariant. While walking up or down, the height estimation should be updated. Luckily, MEMS-IMU has the ability to estimate the ground mode precisely because although the error accumulated, the short-time results of IMU are accurate. The accumulative error of height estimation is shown in Figure 18. We can see that only 5000 sampling points later (25 s), the height error is 2 m which is unacceptable. However, we can see that, in Figure 18b, the maximum height difference every one second is 0.11 m. It stays steady and is possible to use this information to aid barometer to estimate the height more precisely.

**Figure 19:**
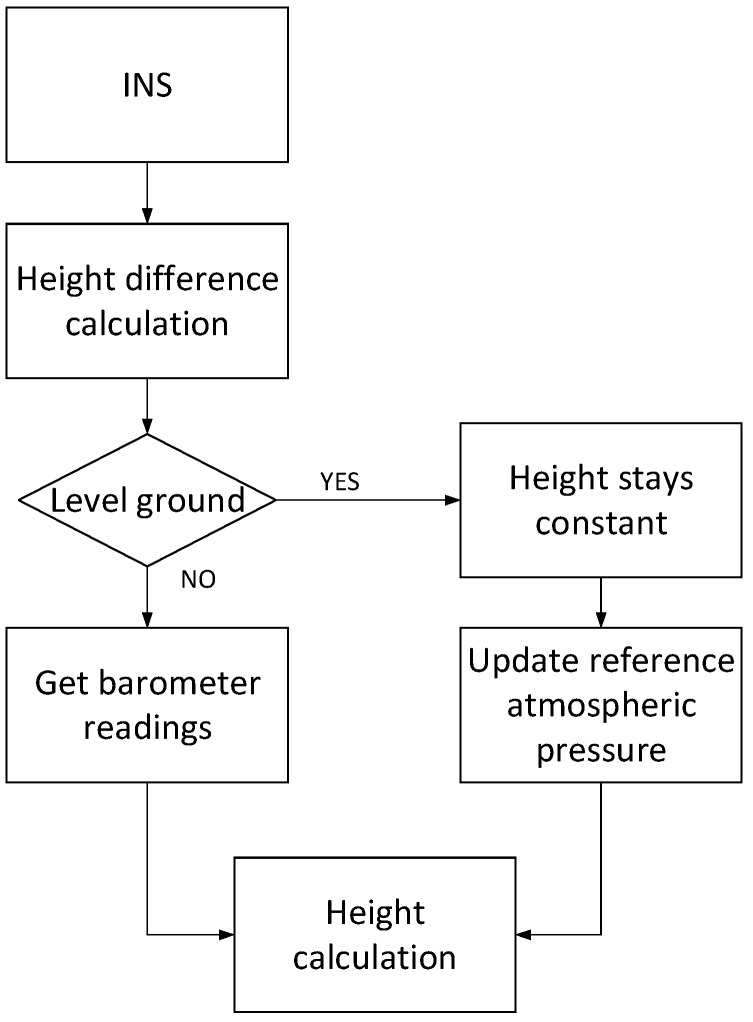
Flowchart of height difference information aided barometer (HDIB) algorithm.

The flowchart of HDIB is shown in Figure 19. It has two stages. The first stage is to distinguish level ground/upstairs/downstairs with MEMS-IMU readings. The second stage is to adaptively set the reference atmospheric pressure according to the result of the first stage. Then difference between the measured atmospheric pressure and the reference atmospheric pressure is used to calculate pedestrians’ vertical position.

We can get accurate height difference between contiguous steps using MEMS-IMU. It can be used to judge whether a person is walking on the level ground, walking upstairs or downstairs. The equations of ground mode estimation are:

(37) $$\left\{ \begin{array}{l} h_{k}=\frac{1}{w}\sum_{n=kw+1}^{kw+w}IMUh_{n}\\ hd_{k}=h_{k}-h_{k-1}\\ \alpha <hd_{k}<\beta \to HDF_{k}=flat\\ hd_{k}<\alpha \to HDF_{k}=down\\ hd_{k}>\beta \to HDF_{k}=up \end{array}\right.$$

where *IMU_h_* is the height estimation result from INS, *w* is the window length for the average calculation, *h* is the mean height calculated from the IMU system, *hd* is the height difference, *k* is the time index, *α* is the down threshold which is a negative number, *β* is the up threshold which is a positive number.

The value of *w* is based on the sampling frequency of MEMS-IMU. Normally *w* = 1/*f* is acceptable. Considering that a person’s leg raises 20 cm during a gait cycle and the average time is set to one second, *α* is set to −0.1 m and *β* is set to +0.1 m.

The second stage is to calculate pedestrians’ vertical position [30]. The ideal atmospheric equation is:

(38) $$P=\rho R_{d}T$$

where *P* is the atmospheric pressure, *ρ* is the air density, *R_d_* is the gas constant whose value is 287.05287 m^2^/(s^2^K), *T* is the thermodynamic temperature of the air. According to the atmospheric static equation, the relationship of atmospheric pressure and height is:

(39) dP=−ρgdH

where *g* is the local gravity, d(·) is the differential calculation, *H* the height. Take (38) to (39):

(40) $$\frac{dP}{P}=-\frac{g}{R_{d}T}dH$$

Ignoring the variation of gravity and temperature, from vertical position *H_r_* to *H_m_*, Equation (40) can be integrated:

(41) $$\int_{P_{r}}^{P_{m}}\frac{dP}{P}=-\int_{H_{r}}^{H_{m}}\frac{g}{R_{d}T}dH$$

where *H_r_* is the reference vertical position, *H_m_* is the vertical position needed to be measured, *P_r_* is the atmospheric pressure of *H_r_*, *P_m_* is the atmospheric pressure of *H_m_*. The calculation result of Equation (41) is:

(42) $$H_{m}=\frac{R_{d}T_{k}}{g}\ln\frac{P_{r}}{P_{m}}+H_{r}$$

where *T_k_* is a simplified temperature integration result of Equation (41) which is an average value of *T_r_* (temperature of *H_r_*) and *T_m_* (temperature of *H_m_*):

(43) $$T_{k}=\frac{T_{r}+T_{m}}{2}$$

**Figure 20:**
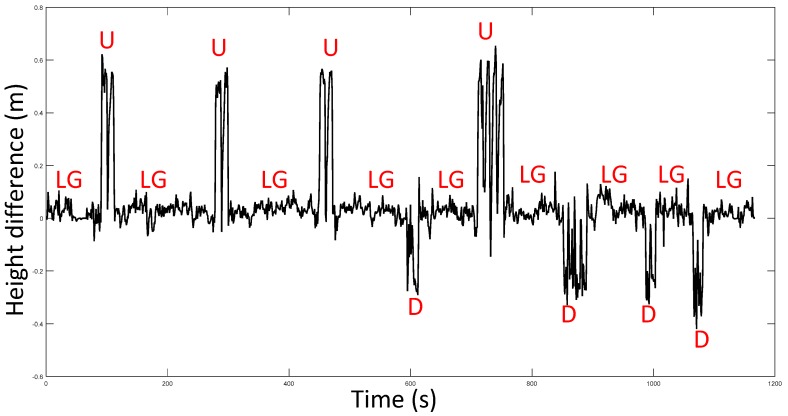
Height difference and the level ground/upstairs/downstairs estimation results. LG is short for level ground. U is short for upstairs. D is short for downstairs.

**Figure 21:**
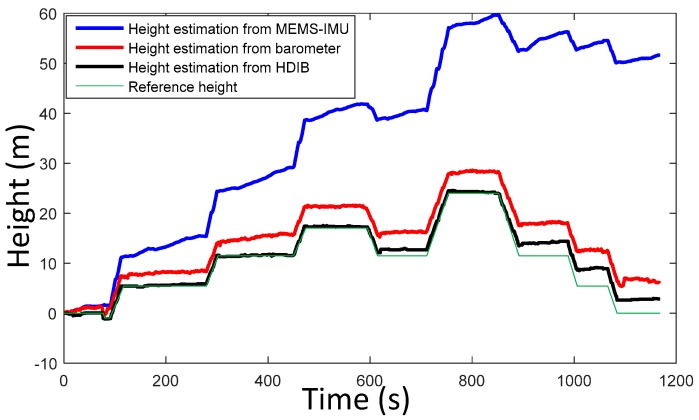
Height estimation using MEMS-IMU, barometer and HDIB. The blue line, red line and black line are height estimation from MEMS-IMU, barometer and HDIB, respectively. The green line is the reference height (true height).

*H_r_* is adaptively adjusted to the result from stage 1 of HDIB. An experiment was taken to test HDIB. The pedestrian started from the second floor, then walked up four floors and went down four floors to return to the start point. This experiment was taken in a shopping mall and the total vertical moving height is around 24 m. The height of start point and end point is 0 m. The sequence of walking is flat, up, flat, up, flat, up, flat, down, flat, up, flat, down, flat, down, flat, down and flat. The estimation result of level ground/upstairs/downstairs is shown in Figure 20 and it matches with the reference height (true height). The positioning results are shown in Figure 21. Three different methods are tested which are MEMS-IMU, barometer and HDIB. Height estimation from MEMS-IMU is the worst because of the accumulative error. The result of using barometer alone fluctuates because of the instability of barometer outputs and the environment. HDIB performs well comparing with the two traditional methods. The height estimation error of the three methods are listed in Table 2.

## 5. A Mag-ZUPT-Based Adaptive Average Window Length Method (ZUPT-AAWL)

TTDE is an important indicator to evaluate the performance of the inertial navigation system. TTDE is defined as the percentage error of the total travelled distance in a closed-loop trajectory:

(44) $$TTDE=\frac{\left\|p(start)-p(end)\right\|}{L}$$

where *p*(*end*) and *p*(*start*) are the end point and start point of the positioning results, ‖⋅‖ is the 2-norm calculation, *L* is the total travelled distance.

Considering that the MEMS-IMU sampling frequency is very high, the original positioning results depict the trajectory of the MEMS-IMU mounted foot (IMU-foot), which is not the same with the walking trajectory. Traditionally, the original positioning results are averaged in a fix length like 1 s because that the gait cycle duration is around 1 s. There is a problem that the averaged results are not one complete gait cycle. It may be a little larger or smaller than the gait cycle according to the length of the average window.

**Figure 22:**
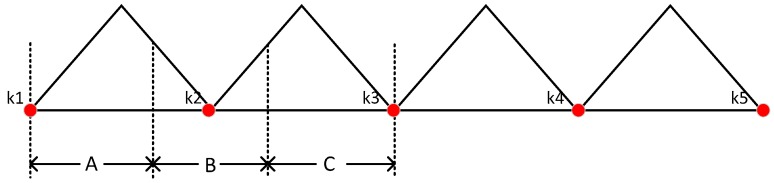
The sketch map of the trajectory of IMU-foot. There are four gait cycles in this figure. Red dots represent that IMU-foot is on the ground which is the mid-stance.

**Figure 23:**
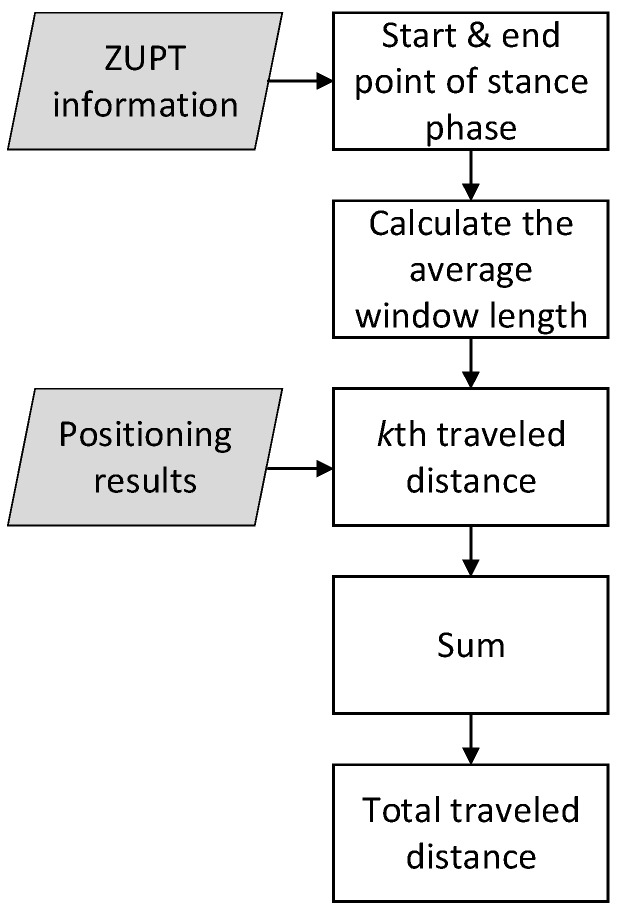
Flowchart of ZUPT-AAWL.

As is shown in Figure 22, during every gait cycle, there must be a time that the IMU-foot is on the ground. It is obvious that $\bar{k_{1}k_{2}}$, $\bar{k_{2}k_{3}}$, $\bar{k_{3}k_{4}}$, and $\bar{k_{4}k_{5}}$ are better choices to be used as the average window length. Because that the length of every gait cycle is different, this window length is adaptive to the specific gait cycle. The flowchart of ZUPT-AAWL algorithm is shown in Figure 23.

Start and end point of stance phase information from ZUPT can be used to determine the average window length:

(45) $$winlen(k)=still_{k}(end)-still_{k}(start)$$

where *winlen*(*k*) is the *k-*th average window length of stance phase, *still_k_*(*end*) is the end point of the *k-*th stance phase and *still_k_*(*start*) is the start point of the *k-*th stance phase. Therefore, the value of average window length is represented in sampling points and has no unit.

The *k-*th trajectory will be averaged with the window length *winlen*(*k*):

(46) $$trajsmooth(k)=\frac{1}{winlen(k)}\times \sum_{i=1}^{winlen(k)}traj_{k}(i)$$

where *trajsmooth*(*k*) is the average length of the *k-*th stance phase, *traj_k_* is the trajectory of the *k-*th stance phase. The trajectory length is calculated using:

(47) $$trajlen(k)=\sqrt{{\left(trajsmooth_{x}(k)-trajsmooth_{x}(k-1)\right)}^{2}+{\left(trajsmooth_{y}(k)-trajsmooth_{y}(k-1)\right)}^{2}}$$

where *trajlen*(*k*) is the *k-*th trajectory length, *trajsmooth_x_* and *trajsmooth_y_* are the *x* and *y* values of *trajsmooth*, respectively.

All the trajectory lengths are summed to calculate the total travelled distance:

(48) L=∑trajlen(k)

**Figure 24:**
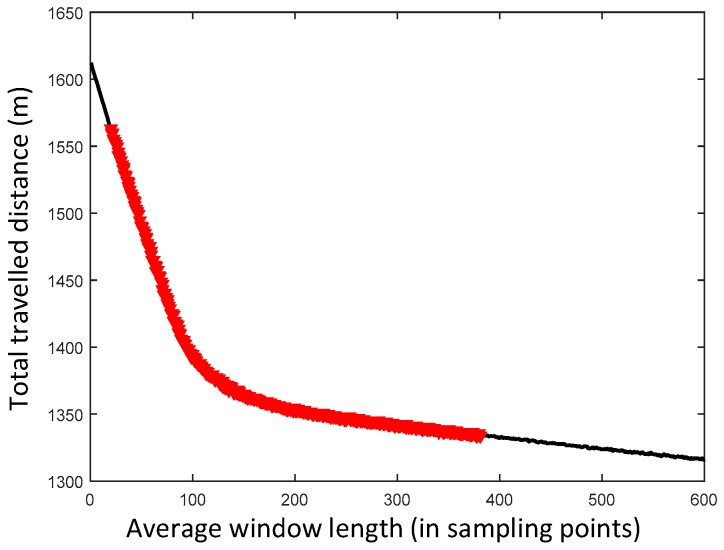
Relationship of total travelled distance and average window length. The red dots are range of ZUPT-AAWL average window length which ranges from 20 to 381 sampling points.

An experiment was performed to verify ZUPT-AAWL. The real total travelled distance measured by a laser range-finder is 1355 m. The relationship of total travelled distance and average window length is shown in Figure 24. The total travelled distance is decreased with the increase of average window length. The red dots are the range of ZUPT-AAWL average window lengths. It ranges from 20 to 381 sampling points according to different gait cycles. The total travelled distance results of four typical fix window length including 1, 100, 200, 300, 400 sampling points and ZUPT-AAWL are shown in Table 3. The result using ZUPT-AAWL is closest to the real length. In addition, ZUPT-AAWL is a more universal method that is adaptive to different trajectories, different moving patterns and different gait cycles.

## 6. Experimental Results

### 6.1. Experimental Setup

**Figure 25:**
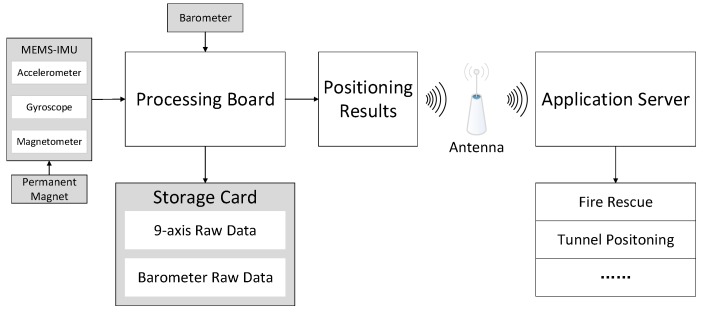
Hardware structure of the 3D positioning system.

In order to evaluate the performance of the 3D positioning system, trajectories of multiple scenarios are tested. The MEMS-IMU used in our system is an AHRS-1 (Inertial Labs, Paeonian Springs, VA, USA), which contains a 3-axis accelerometer, a 3-axis gyroscope and a 3-axis magnetometer. The MEMS-IMU is mounted on the heel of the left shoe and one permanent magnet is mounted on the right shoe. The diameter and thickness of the permanent magnet used in the experiment is 2.5 cm and 1 cm, respectively. A processing board is also mounted on the left shoe. Furthermore, a BMP280 barometer (Bosch, Stuttgart, Baden-Württemberg, Germany) is installed on the processing board. The raw data and processed positioning results are saved in the storage card of the processing board. The 9-axis data obtained from the sensor is processed on board to get the pedestrian positioning results and then transmitted to the server in real-time. In order to evaluate the performance more carefully, in this paper, the raw 9-axis data is processed on the MATLAB platform. The system hardware structure is shown in Figure 25.

The experiments are designed to have a full test of the system. Firstly, three walking gait pattern, three running gait pattern and three walking/running mixed trajectories are taken to test the mag-ZUPT performance. The evaluation indicator of this experiment is the accuracy rate of stance phase estimation. Secondly, nine different ascending/descending stairs experiments are taken to test HDIB algorithm. Finally, six 3D trajectories are taken to evaluate the general performance of this system. In order to get an accurate TTDE, ZUPT-AAWL is used to calculate the total walking distance.

### 6.2. Mag-ZUPT Experiments

Nine tests are taken on the 100 m runway of a stadium. Three walking gait pattern, three running gait pattern and three walking/running mix trajectories are taken to test the mag-ZUPT performance. The true stance phase number is countered by a tester recording the steps. Stance phases of SHOE (shown in Equation (33)) and mag-ZUPT are calculated from the number of 1 in ZUPT.

The results of the nine tests are listed in Table 4. Stance phase estimation performance of walking gait pattern is similar because that in the low dynamic movement, acceleration and angular rate are relatively stable. As to the running gait pattern, the stance phase estimation error of SHOE is serious because that the threshold is adjusted to walking, while mag-ZUPT method has good performance. In the walking/running tests, the subject walks and runs for several steps in turn. Considering that there are some walking steps, stance phase estimation of SHOE is better than the running tests.

The average stance phase number of the nine tests is 50.3. The average error of SHOE and mag-ZUPT is 13.9/50.3 and 0.3/50.3, respectively. The mag-ZUPT method can get a much better stance phase estimation accuracy and ZUPT estimation in multiple moving patterns.

**Figure 26:**
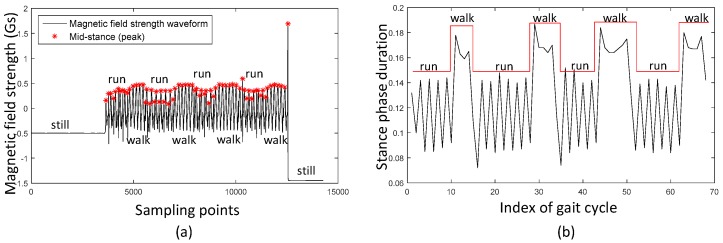
Mid-stance and stance phase duration of Mix_1; (**a**) is magnetic field strength waveform and mid-stances (peaks) extracted from this wave; (**b**) is stance phase duration with different gait cycles. Peak-peak information can represent movement patterns including walking and running. Stance phase duration is calculated according to peak-peak information.

Furthermore, the mistake of stance phase estimation leads to the positioning result divergence. Table 5 shows the total travelled distance of SHOE and mag-ZUPT. It is closely connected with the stance phase estimation result. Choosing Running_3 as an example, the stance phase estimation of SHOE is only 4/37 which leads to a total travelled distance estimation of 686.4/100. The average total moving length average error of SHOE is 100.4/100 comparing 2.3/100 of mag-ZUPT. As an example stance phase duration calculated using Equation (36) and Figure 14 of Mix_1 is shown in Figure 26. It is obvious that peak-peak information can be used to distinguish movement patterns including walking and running. Stance phase duration should be adjusted with different gait cycles.

### 6.3. HDIB Experiments

Six typical trajectories including walking on the level ground, ascending stairs and descending stairs are taken to evaluate HDIB performance. Description of the test trajectories are shown in Table 6. Height_1 and Height_2 are walking on the level ground indoor and outdoor, respectively. Height_3–Height_6 are walking indoor.

Height estimation error root mean square (RMS) is utilized to evaluate the results of the six trajectories, which is shown in Table 7. We can get the following conclusions from the results:

① Barometer based height estimation method is heavily influenced by the environment. Outdoor environment is more complex than indoor. RMS of Height-1 (indoor) is 1.18 m comparing with 0.19 m of Height_2 (outdoor). Generally speaking, height estimation error of barometer is from 1 m to 2 m which is about half of the floor height.
② MEMS-IMU based height estimation method has a large error. However, the short-time accuracy of MEMS-IMU is high and invulnerable to environment factors. Height_5 is a short trajectory with only one floor up and down and MEMS-IMU based method is even better than barometer.
③ HDIB gets the best accuracy among all the six tests.

**Figure 27:**
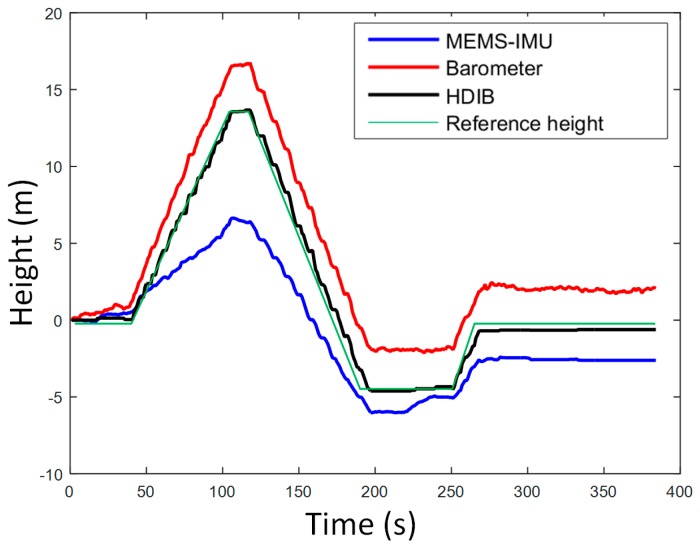
Height estimation results of MEMS-IMU, Barometer and HDIB of Height_4 trajectory. The blue line, red line and black line are height estimation from MEMS-IMU, barometer and HDIB, respectively. The green line is the reference height (true height).

As an example, height estimation results of MEMS-IMU, barometer and HDIB of Height_4 trajectory is shown in Figure 27. RMS of MEMS-IMU, barometer and HDIB is 4.28, 1.78 and 0.69 m, respectively. It is obvious that we can get a more accurate height estimation result using HDIB.

### 6.4. Comprehensive Pedestrian Trajectory Experiments

**Figure 28:**
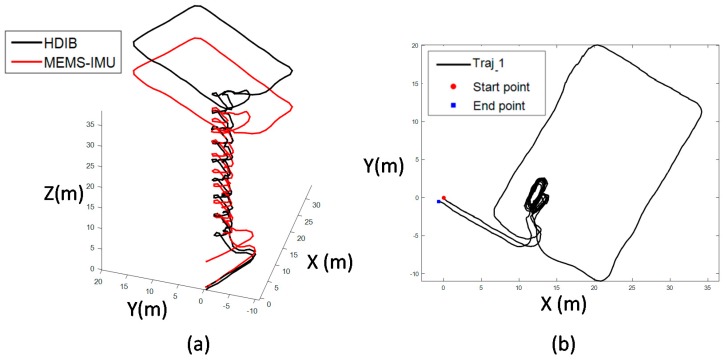
Positioning result of Traj_1; (**a**) is the 3D view of the positioning result; (**b**) is the top view of the positioning result. TTDE of Traj_1 is 0.32% and the height error is 0.68 m.

Six comprehensive experiments are taken to evaluate the 3D positioning system. The key indicators including height error and TTDE are summarized in Table 8. Traj_1 is ascending stairs from floor 2 to floor 11, then walking around a rectangle in floor 11. Finally, the pedestrian returns to the starting point. The total travelled distance of Traj_1 is 261 m with 18 floors up and down. This scenario is mainly to verify the height estimation promotion of HDIB. The 3D positioning result is shown in Figure 28. TTDE of Traj_1 is 0.32% and the height error is 0.68 m.

**Figure 29:**
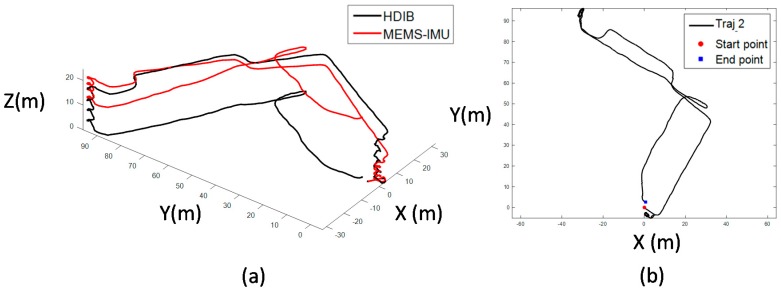
Positioning result of Traj_2; (**a**) is the 3D view of the positioning result; (**b**) is the top view of the positioning result. TTDE of Traj_2 is 0.76% and the height error is 0.27 m.

Traj_2 is a mix of walking on the flat, ascending and descending the stairs. The total travelled distance is 368 m with 5 floor up/down. The 3D and top view of Traj_2 positioning result is shown in Figure 29. TTDE of Traj_2 is 0.76% and the height error is 0.27 m.

**Figure 30:**
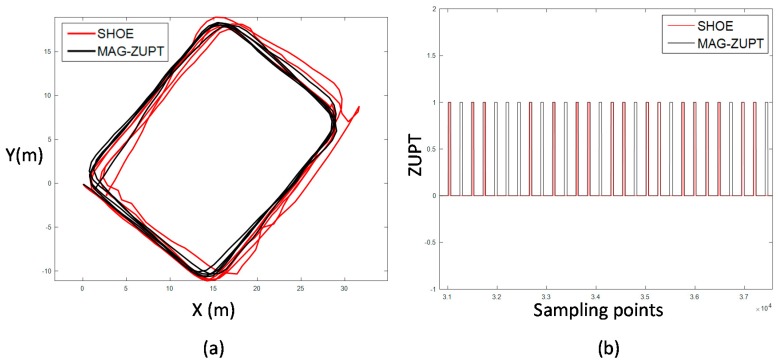
Positioning and ZUPT result of traditional ZUPT and mag-ZUPT of Traj_3; (**a**) is the positioning result. The trajectory of mag-ZUPT is more matched to the true trajectory; (**b**) is ZUPT information. Mag-ZUPT based stance phase estimation has a better performance. TTDE of this test is 0.39%.

Traj_3 is a walking/running mix trajectory along a rectangle of 5 loops. The positioning result is shown in Figure 30a and ZUPT results are shown in Figure 30b. The stance phase estimation of SHOE is less than the real number which leads to the divergence of the positioning result. While the stance phase estimation of mag-ZUPT performs well and the accumulative error is efficiently eliminated. TTDE of Traj_3 is 0.39%.

**Figure 31:**
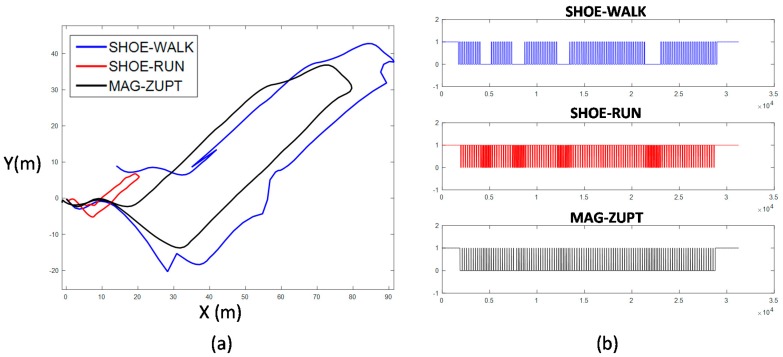
Positioning results and ZUPT comparison of Traj_4; (**a**) is the positioning result. Blue line and red line are the results of SHOE-ZUPT method whose threshold is adjusted to walking and running, respectively. Black line is the result of the novel mag-ZUPT method developed in this paper; (**b**) is the ZUPT result of SHOE-WALK, SHOE-RUN and mag-ZUPT. TTDE of mag-ZUPT is 0.77%.

Traj_4 is a walking and running mix trajectory which is divided to five walking segments and four running segments. The positioning results and ZUPT comparison of SHOE and mag-ZUPT are shown in Figure 31. Considering that SHOE is threshold based which is different in walking and running gait patterns. In this experiment, SHOE-WALK and SHOE-RUNNING are adjusted to the walking and running gait patterns, respectively. The threshold of SHOE-WALK is smaller than SHOE-RUN. If the positioning system applies SHOE-WALK, the calculated trajectory will be longer than the real because more stance phases are misjudged to swing phases. And if SHOE-WALK is applied, the result is contrary to the former one. More swing phases are misjudged to stance phases and the calculated trajectory is much shorter. However, mag-ZUPT is not threshold based and it will self-adjust according to the mid-stance and peak-peak period. Therefore, the TTDE of mag-ZUPT is 0.77% which is much better than SHOE.

**Figure 32:**
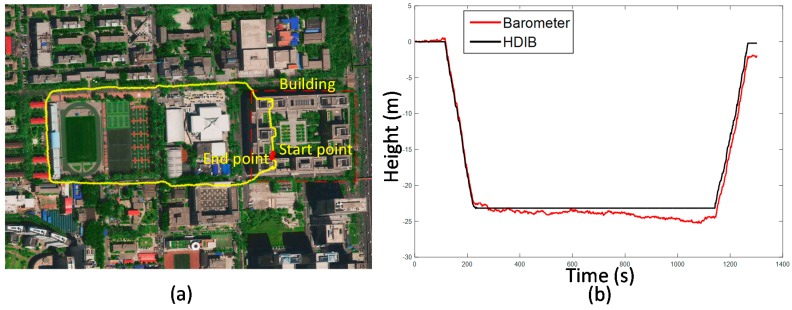
Positioning result of Traj_5 overlaid on a satellite campus picture; (**a**) is the top view; (**b**) is height estimation result. TTDE of this test is 0.63% and the height error is 0.20 m.

**Figure 33:**
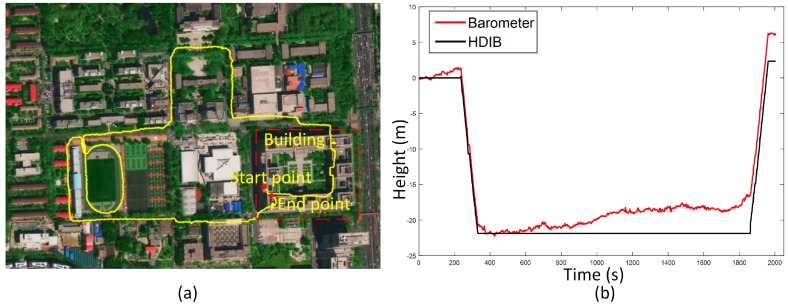
Positioning result of Traj_6 overlaid on a satellite campus picture; (**a**) is the top view; (**b**) is height estimation result. TTDE of this test is 1.04% and the height error is 2.35 m.

Traj_5 and Traj_6 are long distance indoor-outdoor tests. The start point is on floor 6. The pedestrian walks down to the outdoor and walks in the campus, then goes up 6 floors to the start point. Traj_6 with one loop along the stadium is more complex than Traj_5. Positioning results of Traj_5 and Traj_6 are shown in Figure 32 and Figure 33, respectively. TTDE of Traj_5 is 0.63% and the height error is 0.20 m. TTDE of Traj_6 is 1.04% and the height error is 2.35 m.The results show that the method described in this paper can obtain a good 3D positioning result of the complex indoor/outdoor trajectory.

## 7. Conclusions

A novel 3D pedestrian navigation method integrating a MEMS-IMU, barometer and permanent magnet is presented in this paper to solve the stance phase detection, height estimation and total travelled distance calculation problems of foot-mounted inertial systems. Stance phase could be misjudged using acceleration and angular rate-based ZUPT detector like SHOE, especially in running. Mag-ZUPT applying magnetic field strength information of the foot-mounted permanent magnet is proposed to estimate stance phase. Stance phase duration is calculated using the proposed model of stance phase duration and peak-peak information. With stance phase duration, mid-stance is expanded to stance phase. HDIB combines the advantages of MEMS-IMU and barometer to provide high accuracy height estimation. This algorithm has two stages which are level ground/ upstairs/downstairs distinction and height calculation. ZUPT-AAWL is proposed using stance phase information in a gait cycle to set the average window length to calculate the total travelled distance more precisely. The biggest difference of ZUPT-AAWL and the traditional algorithm is that the average window length is adaptively changed according to different gait cycles.

Three kinds of experiments are taken to evaluate performance of the proposed method. The first one includes nine tests of walking/running/mix trajectories. It aims to compare the stance phase detection performance of SHOE and mag-ZUPT. The results show that mag-ZUPT is much better, especially in running. The second one has six tests which include walking on a level ground, ascending stairs and descending stairs to evaluate HDIB performance. The results show that RMS of height estimation of the six tests are all less than one meter, which is much better than MEMS-IMU and barometer alone. The last one contains six comprehensive trajectories including indoor/outdoor, walking/running, ascending/descending stairs. Numerical results show that TTDE of these experiments ranges from 0.32% to 1.04% in both walking and running gait patterns, and the height estimation error is from 0 m to 2.35 m. This 3D positioning method can obtain a good height and TTDE accuracy in both walking and running gait patterns. In our future work, we will use low-cost MEMS-IMU to achieve a similar TTDE performance. More experiments will be taken to establish a more accurate model of stance phase duration and peak-peak period information.

## Figures and Tables

**Table 1:** Statistics of peak-peak time period of walking and running gait patterns.

Items	Peak-Peak Period of Walking	Peak-Peak Period of Running
Maximum	277	156
Minimum	242	141
Average	259.4	148.1

**Table 2:** Height estimation result of MEMS-IMU, Barometer and HDIB.

Method	Start & End (m)	Peak (m)	Final Height (m)	Height Error (m)
MEMS-IMU	0	24	51.72	51.72
Barometer	0	24	5.98	5.98
HDIB	0	24	2.04	2.04

**Table 3:** Typical total travelled distance results.

Average Window Length	Total Travelled Distance (m)
Real length	1355
1	1612
100	1394
200	1353
300	1342
400	1332
ZUPT-AAWL	1356

**Table 4:** Stance phase estimation accuracy of SHOE and mag-ZUPT.

Trajectory	Stance Phase Number	Stance Phase Number of SHOE	Stance Phase Number of Mag-ZUPT
Walking_1	70	69	70
Walking_2	68	69	68
Walking_3	67	66	67
Running_1	39	14	39
Running_2	38	14	37
Running_3	37	4	36
Mix_1	45	34	45
Mix_2	45	32	44
Mix_3	44	28	44
Average error	None	13.9/50.3	0.3/50.3

**Table 5:** Total travelled distance of SHOE and mag-ZUPT.

Trajectory	Actual Moving Length	Moving Length of SHOE	Moving Length of Mag-ZUPT
Walking_1	100	94.8	98.5
Walking_2	100	95.6	98.6
Walking_3	100	94.4	97.2
Running_1	100	176.9	96.6
Running_2	100	185.9	98.2
Running_3	100	686.4	104.7
Mix_1	100	104.8	102.1
Mix_2	100	153.8	98.1
Mix_3	100	180.5	101.5
Average error	None	100.4/100	2.3/100

**Table 6:** Description of the test trajectories.

Trajectory	Height Change Description
Height_1	Walking on the level ground for 396 m, the height change is 0 m.
Height_2	Walking on the level ground for 408 m, the height change is 0 m.
Height_3	Height of each floor is 3.55 m, ascending 4 floors, descending 5 floors, ascending 1 floor
Height_4	Height of each floor is 3.55 m, ascending 4 floors, descending 5 floors, ascending 1 floor
Height_5	Height of each floor is 3.55 m, descending 1 floor and ascending 1 floor
Height_6	Walking from floor 2 to floor 11, height from floor 2 to 7 is 4.108 m, height from floor 7 to floor 11 is 3.792 m

**Table 7:** RMS results of IMU based height estimation, barometer based height estimation and HDIB based height estimation.

Trajectory	Total Height Change (m)	IMU Height Error RMS (m)	Barometer Height Error RMS (m)	HDIB Height Error RMS (m)
Height_1	0	14.15	0.19	0
Height_2	0	22.07	1.18	0
Height_3	35.5	10.29	1.31	0.26
Height_4	35.5	4.28	1.78	0.69
Height_5	7.1	0.80	0.96	0.27
Height_6	35.7	16.94	1.07	0.30

**Table 8:** Key indicators of the six experiments.

Trajectory	Total Moving Distance (m)	Height Error (m)	TTDE
Traj_1	261	0.68	0.32%
Traj_2	368	0.27	0.76%
Traj_3	409	0	0.39%
Traj_4	202	0	0.77%
Traj_5	1430	0.20	0.63%
Traj_6	2696	2.35	1.04%
